# Method for inducing experimental pneumococcal meningitis in outbred mice

**DOI:** 10.1186/1471-2180-4-36

**Published:** 2004-09-22

**Authors:** Damiana Chiavolini, Sergio Tripodi, Riccardo Parigi, Marco R Oggioni, Elisabetta Blasi, Marcella Cintorino, Gianni Pozzi, Susanna Ricci

**Affiliations:** 1 Dipartimento di Biologia Molecolare, Laboratorio di Microbiologia Molecolare e Biotecnologia (LA.M.M.B.), Policlinico "Le Scotte" (5° lotto), Università di Siena, Viale Bracci, 53100 Siena, Italy; 2 Current address: Evans Medical Research Center, 650 Albany Street, Boston University School of Medicine, Boston Medical Center, Boston, MA 02118, USA; 3 Istituto di Anatomia Patologica e Istologia, Policlinico "Le Scotte", Università di Siena, Viale Bracci, 53100 Siena, Italy; 4 Dipartimento di Scienze Igienistiche, Microbiologiche e Biostatistiche, Università di Modena e Reggio Emilia, Modena, Via Campi 287, 41100 Modena, Italy

## Abstract

**Background:**

*Streptococcus pneumoniae *is the leading cause of bacterial meningitis. Pneumococcal meningitis is associated with the highest mortality among bacterial meningitis and it may also lead to neurological sequelae despite the use of antibiotic therapy. Experimental animal models of pneumococcal meningitis are important to study the pathogenesis of meningitis, the host immune response induced after infection, and the efficacy of novel drugs and vaccines.

**Results:**

In the present work, we describe in detail a simple, reproducible and efficient method to induce pneumococcal meningitis in outbred mice by using the intracranial subarachnoidal route of infection. Bacteria were injected into the subarachnoid space through a soft point located 3.5 mm rostral from the bregma. The model was tested with several doses of pneumococci of three capsular serotypes (2, 3 and 4), and mice survival was recorded. Lethal doses killing 50 % of animals infected with type 2, 3 and 4 *S. pneumoniae *were 3.2 × 10, 2.9 × 10 and 1.9 × 10^2 ^colony forming units, respectively. Characterisation of the disease caused by the type 4 strain showed that in moribund mice systemic dissemination of pneumococci to blood and spleen occurred. Histological analysis of the brain of animals infected with type 4 *S. pneumoniae *proved the induction of meningitis closely resembling the disease in humans.

**Conclusions:**

The proposed method for inducing pneumococcal meningitis in outbred mice is easy-to-perform, fast, cost-effective, and reproducible, irrespective of the serotype of pneumococci used.

## Background

Bacterial meningitis is an important infection of the central nervous system (CNS), and the three major responsible bacteria are *Neisseria meningitidis*, *Haemophilus influenzae *and *Streptococcus pneumoniae *[1]. Despite the use of antimicrobial therapy, pneumococcal meningitis (PM) has the highest case-fatality rate (up to 30 %) for bacterial meningitis, and in 27 % of cases, it leads to serious neurological sequelae, including cognitive impairment [2,3]. Development of PM generally starts from pneumococcal colonisation of the nasopharynx, which is the natural reservoir of *S. pneumoniae *in humans and especially in children [4]. The pathogenic steps leading to PM include invasion of the bloodstream from the nasopharyngeal mucosa, survival in the blood, and subsequent entry into the CNS by crossing the blood-brain-barrier (BBB) [1,2]. However, PM can also be caused by either contiguous spread of pneumococci infecting the sinuses or the middle ear, or accidental traumatic inoculation of bacteria into the CNS [2]. A recent paper showed that non-hematogenous invasion of the brain by *S. pneumoniae *in mice may also occur through retrograde axonal transport along olfactory neurons [5]. Once the pneumococcus starts replicating in the cerebrospinal fluid (CSF), severe inflammation occurs in cerebral vessels and subarachnoid space, and damage to the brain parenchyma is produced [1,2].

Animal models of PM have been developed in order to: (i) characterise the pathogenesis of meningitis, (ii) analyse the role of pneumococcal virulence factors in the disease, (iii) understand the host immune response to *S. pneumoniae *infection, and (iv) test the efficacy of novel antibiotics and vaccine candidates. Both infant and adult rats [6-9], and also adult rabbits [10-13] have largely been employed as animal models to characterise PM induced by intracisternal inoculation of bacteria. Infant rats have also been used to study haematogenous meningitis following intraperitoneal infection with *S. pneumoniae *[14]. However, models of PM have also been developed in the mouse by using the following routes of infection: (i) intraperitoneal (i.p.) [15-17], (ii) intranasal (i.n.) [18,19], or (iii) intracranial (i.c.) parenchymal [20,21] or cisternal [22,23]. Haematogenous murine meningitis models (both i.p. and i.n.) allow to study PM pathogenesis, and i.n. models are particularly useful as they mimic the natural infection route of *S. pneumoniae *in humans. However, those models present the disadvantage that PM is induced in about half of the animals, while the remaining mice may die of sepsis without developing meningitis. Models of meningitis induced by the i.p. route were employed to carry out therapeutic studies [16] and investigations on PM pathogenesis [15,17]. The i.n. model by Zwijnenburg *et al. *[19] was employed in interleukin (IL)-10, IL-18, and IL-1 receptor deficient mice to investigate the role of different cytokines in PM [24-26]. Direct induction (i.c. route) of PM mimics meningitis caused by contiguous spread from the sinuses or traumatic entrance of pneumococci into the CNS and allows to study the host-parasite interaction in the brain. Besides an early model of i.c.-induced meningitis in mice [20] employed in therapeutic studies [6,20], the model by Gerber *et al*. is an useful and reliable system for causing PM in the mouse [21]. Following infection of C57BL/6 inbred mice into the right lobe of the brain with type 3 pneumococci, bacterial enumeration in different organs, brain histology, behavioural tests, and clinical scores were performed [21]. A model of intracisternal infection was described by Koedel *et al*., who induced meningitis via inoculation of *S. pneumoniae *(type 3) into the *cisterna magna *of C57BL/6 mice and investigated the function of nitric oxide in the disease [23]. Both models, largely employed in studies on the roles of both pneumococcal [27] and host factors [28-32] in PM, rely on the use of inbred mouse strains and type 3 pneumococci.

In the present study, we describe an experimental model of PM in outbred mice based on the direct inoculation of bacteria into the subarachnoid space through a soft point located 3.5 mm rostral from the bregma. Both the technique employed for infection and the anatomical coordinates of the inoculation site are accurately described. The model was tested with pneumococcal strains of three different capsular serotypes and it was characterised in detail by using TIGR4 (type 4) as a model strain. The proposed method is precise, simple, cost-effective, fast and reproducible, and the disease induced closely resembles PM in humans.

## Results

### Inoculation site and technique

**Figure 1:**
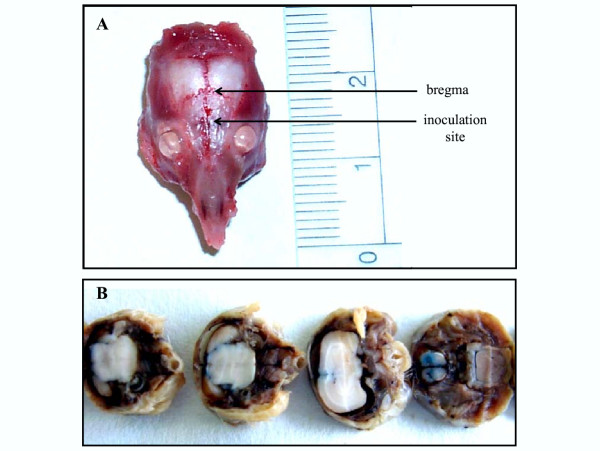
**Site of injection in mice inoculated via the i.c. subarachnoidal route. **Three MF1 mice were injected with 30 μl of trypan blue via the i.c. subarachnoidal route through a soft point located 0 mm lateral, 3.5 mm rostral and 2 mm ventral from the bregma. After 30 minutes, animals were sacrificed and decapitated. Their skulls were fixed in formalin, decalcified and then sectioned. Results from one mouse are shown. **A. **The exact location of the inoculation site with respect to the bregma is indicated by an arrow. Location of the bregma is also shown. **B. **Mouse brains were cut in correspondence of the site of injection and then sectioned into coronal planes. Diffusion of trypan blue from the inoculation site into the subarachnoid and ventricular spaces is visible.

The bregma is the intersection of the coronal and sagittal sutures of the skull and can be recognised in mice by visual examination of the exposed skull (Fig. 1A). We inoculated mice by the i.c. route through a soft point located along the skull midline 3.5 mm rostral to the bregma (Fig. 1A). The stereotaxic coordinates of such inoculation point are 0 mm (× plane), 3.5 mm rostral (y plane) and 2 mm ventral (z plane) from the bregma [33]. A preliminary study was carried out to trace diffusion of the inoculation material from the point of injection into the brain. Three animals were injected with 30 μl of trypan blue and sacrificed 30 minutes after inoculation. Following decapitation, skulls were sectioned into coronal planes, and diffusion of trypan blue was observed (Fig. 1B). The dye rapidly spread from the injection site into the subarachnoid and ventricular spaces (Fig. 1B); hence, this infection route is referred to as i.c. subarachnoidal. Histological analysis of the brain sections confirmed that the inoculation needle crossed the mouse frontal lobes and reached the subarachnoid space (data not shown). In order to assess whether the inoculation technique was traumatic to animals, another experiment was performed by inoculating three control mice with saline. Animals recovered soon after injection and did not present any neurological problem (*i.e. *lethargy, paralysis) for several weeks after inoculation (data not shown).

These data allowed localisation of the anatomical coordinates of the inoculation site and proved the suitability of the i.c. subarachnoidal infection technique.

### Meningitis induction by type 2, 3 and 4 *S. pneumoniae*

After characterisation of the inoculation site and technique, mice were infected with pneumococci, and the establishment of PM was evaluated and clearly evidenced by histological analysis (see below).

In order to test the model with different pneumococcal serotypes, dose-dependent survival studies were performed, and the lethal doses killing 50% of animals (LD_50_) were calculated. We chose three commonly used *S. pneumoniae *strains, such as D39, HB565, and TIGR4. The D39 strain (type 2) is the encapsulated parent of the rough type 2 R36A strain used by Avery [34-36]. The HB565 strain (type 3) is a streptomycin-resistant derivative of the A66 strain used by Avery [34,36,37]. The serotype 4 TIGR4 is the genome strain sequenced by the Institute for Genomic Research [38]. The survival patterns of mice inoculated with D39 and HB565 were almost identical, with LD_50 _of 3.2 × 10 and 2.9 × 10 colony forming units (CFU), respectively (data not shown). The TIGR4 strain was less virulent in the i.c. subarachnoidal infection model compared to D39 and HB565, as its LD_50 _was 1.9 × 10^2 ^CFU (data not shown).

### Animal survival and bacterial titres after infection with the TIGR4 strain

**Figure 2:**
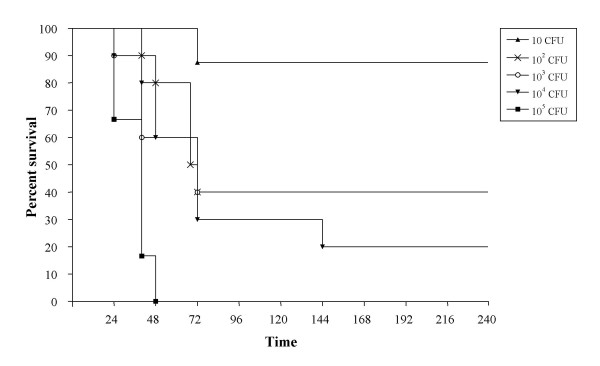
**Kaplan-Meyer survival curve of mice infected with type 4 *S. pneumoniae***. Five groups of MF1 mice (n = 6–10) were infected by the i.c. subarachnoidal route with different doses of type 4 *S. pneumoniae *ranging from 10 to 10^5 ^CFU per mouse. Mice were monitored for 10 days and survival was recorded. Results are expressed as percentage of survival over time.

To describe the features of PM in detail following i.c. subarachnoidal infection of mice, we chose to characterise PM induced by the TIGR4 genome strain. For this purpose, we performed time-dependent survival studies, bacterial counts in different organs/tissues, and histological analysis of brain and spleen (see below). In order to study animal survival, five groups of MF1 mice were infected with doses of TIGR4 increasing from 10 to 10^5 ^CFU per mouse. The percentage of animals surviving over time at each bacterial dose was analysed by a Kaplan-Meier curve (Fig. 2). After inoculating 10 CFU, only one mouse out of eight died 72 hours after infection (87.5 % survival). At the doses of 10^2 ^CFU and 10^3 ^CFU, 40 % of mice survived pneumococcal challenge, whereas the remaining 60 % died within 72 hours from infection. Survival further decreased from 20 % in mice infected with 10^4 ^CFU to 0 % following infection with 10^5 ^CFU, which induced severe symptoms and subsequent death of all mice within the first 48 hours after challenge (Fig. 2). The median time-to-death of the groups injected with 10, 10^2^, 10^3^, 10^4^, and 10^5 ^CFU were 240, 70, 72, 72, and 40 hours, respectively. From these results, the median survival time of animals did not vary at intermediate doses (10^2^, 10^3^, 10^4 ^CFU), while it considerably decreased at the highest dose (10^5 ^CFU) leading to rapid death of all mice.

To determine the number of pneumococci in brain, spleen and blood at the final stages of the disease, moribund mice infected with 10^5 ^CFU of TIGR4 were sacrificed, samples collected and appropriately treated, and viable counts were carried out. Moribund animals showed comparable bacterial counts in the brain (3.1 × 10^6 ^± 1.3 × 10^6 ^CFU/organ). Similarly, dissemination from the brain to vital organs occurred and was consistent in all animals, with bacterial counts of 3.8 × 10^6 ^± 4.8 × 10^6 ^CFU and 2.1 × 10^8 ^± 3.0 × 10^8 ^CFU in the spleen and blood, respectively (data not shown).

### Histological characterisation of the PM model

In order to prove the establishment of PM and study the features of the disease, we performed histological analysis on the brain of moribund mice inoculated with several doses of the TIGR4 strain and sacrificed at various time-points after infection. Animals at the final stages of the disease showed typical signs of meningitis (*i.e. *hunchbacked, photophobic, lethargic), but they did not develop hemiparesis or plegia. Moribund mice that had received 10^2^, 10^3^, 10^4^, and 10^5 ^CFU were humanely killed at 72, 72, 48, and 24 hours post-infection, respectively. Two animals for each pneumococcal dose and three additional control mice were sacrificed. Brains and spleens were excised and treated for both haematoxilin-eosin and Gram staining.

**Figure 3:**
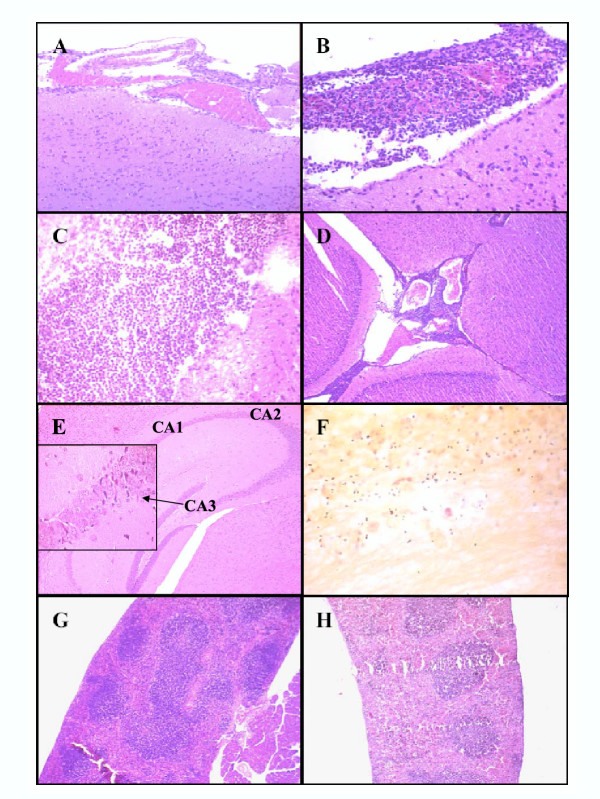
**Histological analysis of the brain and spleen of mice infected with type 4 *S. pneumoniae***. MF1 outbred mice were infected by the i.c. subarachnoidal route with the TIGR4 strain and humanely killed before reaching the moribund state. Brains (A-F) and spleens (G-H) were excised, fixed in formalin, embedded in paraffin, and stained with either haematoxilin-eosin or Gram. **A. **Mild inflammatory changes with congested leptomeningeal blood vessels and PMNs margination. **B-C. **Severe inflammation characterised by cellular exudates composed of PMNs entrapped in fibrin in the subarachnoid space. In panel C, the fibrin web is clearly visible. **D. **Acute inflammation in the ventricular spaces. **E**. Brain damage in the hippocampus: neuronal shrinkage in the CA3 hippocampal region is shown in the inset. The location of CA1, CA2 and CA3 areas is represented. **F. **Gram staining of pneumococci in the subarachnoid space of the brain of moribund mice. Short chains (mainly diplococci) of Gram positive bacteria surrounded by granulocytes. **G-H. **Haematoxilin-eosin stained spleen sections. A distinct congestion of the red pulp together with considerable modifications of the white pulp are present in the spleen of animals infected with *S. pneumoniae *(G) compared to controls (H).

In infected mice, no cerebral abscesses were observed, but only granulocytic infiltrations involving the subarachnoid and ventricular spaces. Differently, control animals injected with saline showed no histological changes following inoculation. Brains of moribund mice following infection with the TIGR4 strain showed different degrees of inflammatory changes. Inflammation was regarded as mild in the presence of marked congestion of leptomeningeal blood vessels with margination of polymorphonuclear cells (PMNs), edema, and wisps of fibrin (Fig. 3A). Inflammation was considered severe when the subarachnoid space (Fig. 3B,3C) and/or the ventricular spaces (purulent ventriculitis) (Fig. 3D) contained cellular exudates composed of PMNs entrapped in a dense fibrin net. No large areas of cerebral necrosis were found; however, in some cases, brain damage represented by neuronal shrinkage was observed in the hippocampus (Fig. 3E). Gram staining of brain sections of infected mice revealed the presence of short chains (mainly diplococci) of Gram-positive dark blue bacteria in the subarachnoid space; pneumococci were located mainly extracellularly in a background of PMNs (Fig. 3F). Analysis of the spleen of animals infected with *S. pneumoniae *revealed histological changes in both the white and red pulp with a massive congestion of the red pulp (Fig. 3G), compared to the spleen of control mice (Fig. 3H). These data demonstrate that the i.c. subarachnoidal route of infection is an effective and reliable way for inducing PM in the mouse.

## Discussion

*S. pneumoniae *is one of the causative agents of bacterial meningitis responsible for death and sequelae worldwide. The mouse has largely proved to be a reliable animal model for studying two major pneumococcal diseases such as pneumonia [39-44] and sepsis [45-47]. In the case of PM, the rat [6-9,14] and the rabbit [10-13] have often been preferred to the mouse. With the exception of an initial work describing an i.c. infection procedure for inducing PM in mice in outbred mice [20], only recently, models of PM based on i.c. inoculation were made available in inbred mice [21,23].

In the present work, starting from an experimental method used to study meningitis caused by *Cryptococcus neoformans *[48], we developed a model of PM based on the inoculation of bacteria into the subarachnoid space of outbred mice. This infection route (i.c. subarachnoidal) mimics bacterial entrance into the CNS from the sinuses or the middle ear, or following a trauma. We chose to inoculate mice into the subarachnoid space through a soft point located on the skull 0 mm lateral, 3.5 mm rostral, and 2 mm ventral from the bregma. Such point easily allows inoculation of bacteria from the skull through the frontal lobes into the subarachnoid space, as shown by a preliminary experiment in which trypan blue was used to localise the injection site and trace the inoculum within the brain (Fig. 1). The finding that the inoculation technique did not cause any trauma to animals can be explained by the fact that frontal lobectomy is tolerated in both humans [49] and rats [50], as frontal lobes are mainly committed to behavioural and cognitive functions. We decided to use MF1 outbred mice because this strain is well-known for its susceptibility to both intranasal [40,51,52] and intravenous [47] challenge with *S. pneumoniae*, and because the use of outbred strains is cost-effective. Another research group had previously used CD-1 outbred mice in a study on the efficacy of clinafloxacin against PM; however, the authors did not provide a detailed description of the model [22].

Our i.c. subarachnoidal infection model was tested by using a range of bacterial doses of three different *S. pneumoniae *strains. The strains chosen are the serotypes routinely employed by researchers in the pneumococcal field, and have proved to be highly pathogenic in different mouse infection models [27,40,47,52]. The use of several bacterial doses is also important, as it allows a more accurate evaluation of virulence for each strain, as well as establishing the most appropriate dose to be employed in different studies. The model proved to be suitable for use with pneumococci of different serotypes, as type 2 D39, type 3 HB565, and type 4 TIGR4 were all able to cause PM with LD_50 _ranging from 2.9 × 10 to 1.9 × 10^2 ^CFU. Then, PM was further characterised and standardised by using TIGR4 as a model strain. Kaplan-Meier survival analysis of animals inoculated with different doses of TIGR4 showed that 10^5 ^CFU was lethal for all mice within 48 hours from infection (Fig. 2), suggesting it as the appropriate dose inducing PM similar to hyperacute meningitis in humans. Moribund mice with acute meningitis after infection with 10^5 ^CFU were also septicaemic, as pneumococci could also be recovered from the blood and spleen. Bacterial titres were consistent in each organ/tissue of every mouse examined, underlining the reproducibility of data obtainable by using our model. Dissemination of pneumococci after infection with lethal doses is also in agreement with other i.c. murine models of PM [21,22].

The actual induction and subsequent characterisation of PM caused by the TIGR4 strain was then carried out by histological analysis of the brain tissue from moribund animals. We chose not to analyse PM by viable counting of pneumococci in the CSF, due to the difficulty of sample collection [53] and to the necessity of using pooled CSF samples [21]. Histological examination of the brain showed both cases of mild meningeal inflammation and cases of severe granulocytic effusion in the subarachnoid and ventricular spaces (purulent ventriculitis) (Fig. 3). Cerebral abscesses were not observed, further confirming that the our i.c. subarachnoidal model is indeed a meningitis and not an encephalitis model of infection. Neither inflammatory changes nor death were observed following injection of saline into control mice. Inflammation and PMNs distribution in the brain of moribund mice closely mimicked the histopathology of meningitis in humans [54]. Post-mortem examination of brains from patients who rapidly (less than 24 hours) died due to hyperacute meningitis generally reveals the presence of mild lesions consisting of a sparse leptomeningeal exudate with vessel congestion and PMN margination, in contrast to patients who survived for two or more days, who often exhibit a severe inflammation with fibrin and PMNs in subarachnoid and ventricular spaces [54]. A detailed analysis of PM largely resembling human meningitis was also reported by Gerber *et al.*, who injected C57BL/6 inbred mice into the right lobe of the brain [21]. In that model, the inoculation site was characterised by a large purulent infiltrate present in both meninges and ventricula, and necrosis was observed in all investigated brain regions [21]. In another model, proposed by Koedel *et al*., pneumococci were given by transcutaneous injection directly into the *cisterna magna *[23]. In that study, brain lesions occurred in all mice 24 hours after infection, and histopathological examination revealed intense granulocytic infiltrations in the subarachnoid and ventricular spaces, and absence of cortical necrosis [23]. This finding differs from the model by Gerber *et al.*, who instead reported the presence of extensive cerebral necrotic processes [21]. In our model, we could not observe large areas of necrosis, but we found some signs of neuronal damage (*i.e. *neuronal shrinkage with picnotic nuclei) in the hippocampus of a few animals.

## Conclusions

The present work proposes a method to induce experimental PM in outbred mice by using an i.c. subarachnoidal route of infection. The stereotaxic coordinates of the injection site are provided to allow easy recognition of the inoculation point in the mouse. The model is simple and fast, and the technique assures the development of meningitis, as demonstrated by histological analysis, survival data, and microbiological parameters. No significant differences were observed in the ability of the three pneumococcal strains used to cause disease, emphasising the value of the model. It is worth noting that the use of outbred mice still results in data reproducibility, as replicates in this model closely paralleled each other in terms of survival, CFU counts per organ, and histopathological features. In addition, experiments in outbred mice are cost-effective and can be performed in larger animal groups thereby improving statistical significance. This experimental PM model may be particularly useful for all researchers involved in studies that will investigate the host-pathogen interaction at the cerebral level, with emphasis on both pathogen-associated virulence factors and host-specific brain defences.

## Methods

### Pneumococcal strains, media and growth conditions

Survival studies were performed with the D39, HB565 and TIGR4 strains. TIGR4 was chosen as a model strain for histological characterisation of PM and CFU counts in organs. *S. pneumoniae *was cultured in Tryptic Soy Broth (TSB, Difco, Detroit, MI) at 37°C with 5 % CO_2_. Solid media were obtained by addition of 1.5 % agar and 3 % defibrinated horse blood (Biotec s.n.c., Grosseto, Italy) to TSB. When necessary, streptomycin was used at the final concentration of 500 μg/ml.

### Mice

Outbred 9-weeks-old female MF1 mice weighing 25–30 grams were obtained from Harlan Nossan (Correzzana, Italy). Animals were allowed to settle in the new environment for one week before performing the experiments, they were caged and given food and water *ad libitum*. All animal experiments were approved by the Local Ethical Committee (document no. 754/03, 12.9.03, see Additional file 1) and were conducted according to institutional guidelines.

### Preparation of the challenge dose

Mouse-passaged *S. pneumoniae *strains were prepared by using a modified version of a previously described method [40]. Briefly, bacteria were injected i.p. into mice and recovered 16 hours later from homogenising the spleens with a screen mesh in 2 ml of ice-cold sterile H_2_O. Passaged bacteria were grown to mid-exponential phase, centrifuged for 20 minutes at 1500 × *g*, resuspended in fresh TSB containing 10 % glycerol, and stored in aliquots at -70°C. Numbers of bacteria were determined by viable counting of serial dilutions in sterile phosphate-buffered saline, pH = 7.4 (PBS), and plating onto blood-agar plates. Before inoculation, bacteria were thawed at room temperature, harvested by centrifugation, and resuspended in sterile PBS at the appropriate dilutions.

### Mouse model of meningitis

PM was induced in lightly anaesthetised mice (50 mg/kg ketamine and 3 mg/kg xylazine) by modifying a method previously used to establish meningitis by *C. neoformans *in mice [48,55]. Animals were immobilised by hand and inoculated i.c. at a depth of about 2 mm through a soft point located 3.5 mm rostral from the bregma. A preliminary experiment was carried out by injecting 30 μl of trypan blue i.c. into three MF1 mice. After 30 minutes, animals were sacrificed and decapitated. Their skulls were fixed in 10 % buffered formalin for 24 hours and treated with Decal (Decal Corporation, Tallman, NY) for 24 hours. Coronal sections of about 3 mm were made, and diffusion of trypan blue was observed. Then, to localise the injection site within the brain, the above sections were embedded in paraffin and treated for histological analysis. Standard experiments were performed by injecting the bacterial inoculum in a total volume of 30 μl. Injections were performed by using glass micro-syringes (Hamilton, Bonaduz, Switzerland) with 26 gauge needles.

### Survival studies

Different bacterial doses ranging from 10 to 10^4 ^CFU per mouse were used to infect mice (n= 4) with strains D39 and HB565. In the case of TIGR4, groups of 6 to 10 animals each were inoculated with doses ranging from 10 to 10^5 ^CFU per animal. Control mice were inoculated with PBS (30 μl). Mice were closely monitored twice a day for clinical symptoms (starry fur, hunched appearance, photophobia, lethargy, moribund). Mice were humanely killed before reaching the moribund state. Survival was recorded for 10 days (240 hours).

### Microbiology and histology

Infected mice were sacrificed either for microbiological or histological analysis. Animals were humanely killed before being moribund, and various samples were collected. For CFU counts, blood was withdrawn by cardiac puncture before sacrifice and added to a tube containing 3.8 % of sodium citrate. Brains and spleens were excised and homogenised in 2 ml of sterile PBS. Bacterial counts in blood, brain and spleen were performed by plating 10-fold dilutions onto blood-agar plates. For histopathological analysis of tissues after infection with TIGR4, brains and spleens were immediately fixed in formalin for 24 hours and then embedded in paraffin according to standard procedures. The brains were entirely sectioned along a coronal plane. Sections were stained with both haematoxilin-eosin and Gram according to standard techniques. Morphological changes were assessed by using routine light microscopy. The presence and degree of inflammation were carefully evaluated.

### Statistical analysis

Calculations of LD_50 _values were performed by using both the method by Reed and Muench [56] and Probit analysis with 95 % confidence interval [57]. Survival over time was analysed by the Kaplan-Meier curve.

## Authors' contributions

DC, animal experiments, microbiological analysis, writing of manuscript. ST, histological analysis, writing of manuscript. RP, animal experiments, microbiological analysis. MRO, experimental design. EB, development of methodology for induction of meningitis. MC, co-ordination of histological analysis. GP, co-ordination and design of the study, data evaluation. SR, co-ordination and design of the study, data evaluation, direct supervision of experimental work, writing of manuscript.

All authors read and approved the manuscript.

## Supplementary Material

Additional File 1 Document stating the ethical approval for animal experimentation conceded to the Laboratory of Molecular Microbiology and Biotechnology (LA.M.M.B.) from the University Hospital of Siena, the Medical Faculty and the Local Ethical Committee (document no. 754/03, 12.9.03). Click here for file
