# Supplementary material for: Method for inducing experimental pneumococcal meningitis in outbred mice
Source: BMC Microbiol. 2004 Sep 22;4:36. doi: 10.1186/1471-2180-4-36 (PMC524167; doi:10.1186/1471-2180-4-36)
Supplement: Additional File 1 — Document stating the ethical approval for animal experimentation conceded to the Laboratory of Molecular Microbiology and Biotechnology (LA.M.M.B.) from the University Hospital of Siena, the Medical Faculty and the Local Ethical Committee (document no. 754/03, 12.9.03). [file 1471-2180-4-36-S1.pdf]

Università degli Studi di Siena
Dipartimento di Biologia Molecolare - Sezione di Microbiologia

Siena, July 28, 2004

TO WHOM IT MAY CONCERN

On the 1st of October 2003, the University Hospital of Siena, the Faculty of Medicine and Surgery, and the Local Ethical Committee gave the authorisation to the Laboratory of Molecular Microbiology and Biotechnology (LA.M.M.B.) to perform *in vivo* experiments based on the use of animal models of infection by *Streptococcus pneumoniae* (Prot. no 754/03, 12.9.03). The use of experimental murine models of pneumococcal pneumonia, sepsis and meningitis in mice was thereby approved. The authorisation allows the use of 600 mice and it is valid until the 30th of September 2006 (36 months).

Sincerely yours,

Prof. Gianni Pozzi
Responsible of LA.M.M.B.
Tel.: +39-0577-233430
E-mail: pozzi@unisi.it

Prof. Gian Maria Rossolini
Department Head
Tel.: +39-0577-233455
E-mail: rossolini@unisi.it
